# ATP Consumption by Sarcoplasmic Reticulum Ca^2+^ Pumps Accounts for 40-50% of Resting Metabolic Rate in Mouse Fast and Slow Twitch Skeletal Muscle

**DOI:** 10.1371/journal.pone.0068924

**Published:** 2013-07-01

**Authors:** Ian Curtis Smith, Eric Bombardier, Chris Vigna, A. Russell Tupling

**Affiliations:** Department of Kinesiology, University of Waterloo, Waterloo, Ontario, Canada; University of Queensland, Australia

## Abstract

The main purpose of this study was to directly quantify the relative contribution of Ca^2+^ cycling to resting metabolic rate in mouse fast (extensor digitorum longus, EDL) and slow (soleus) twitch skeletal muscle. Resting oxygen consumption of isolated muscles (VO_2_, µL/g wet weight/s) measured polarographically at 30^°^C was ~20% higher (P<0.05) in soleus (0.326 ± 0.022) than in EDL (0.261 ± 0.020). In order to quantify the specific contribution of Ca^2+^ cycling to resting metabolic rate, the concentration of MgCl_2_ in the bath was increased to 10 mM to block Ca^2+^ release through the ryanodine receptor, thus eliminating a major source of Ca^2+^ leak from the sarcoplasmic reticulum (SR), and thereby indirectly inhibiting the activity of the sarco(endo) plasmic reticulum Ca^2+^-ATPases (SERCAs). The relative (%) reduction in muscle VO_2_ in response to 10 mM MgCl_2_ was similar between soleus (48.0±3.7) and EDL (42.4±3.2). Using a different approach, we attempted to directly inhibit SERCA ATPase activity in stretched EDL and soleus muscles (1.42x optimum length) using the specific SERCA inhibitor cyclopiazonic acid (CPA, up to 160 µM), but were unsuccessful in removing the energetic cost of Ca^2+^ cycling in resting isolated muscles. The results of the MgCl_2_ experiments indicate that ATP consumption by SERCAs is responsible for 40–50% of resting metabolic rate in both mouse fast- and slow-twitch muscles at 30^°^C, or 12–15% of whole body resting VO_2_. Thus, SERCA pumps in skeletal muscle could represent an important control point for energy balance regulation and a potential target for metabolic alterations to oppose obesity.

## Introduction

Skeletal muscle represents ~40% of body weight and accounts for ~20 to 30% of whole body basal metabolic rate [1,2]. Like other cell types, a portion of basal metabolic rate in skeletal muscle can be attributed to the cycling of calcium ions across cell membranes but very few studies have attempted to quantify the relative contribution of Ca^2+^ cycling to resting muscle metabolism. Under basal conditions in skeletal muscle, sarco(endo) plasmic reticulum Ca^2+^-ATPases (SERCAs) are responsible for maintaining a >10^4^-fold Ca^2+^ concentration gradient across the sarcoplasmic reticulum (SR) membrane and for keeping the cytosolic free Ca^2+^ concentration ([Ca^2+^]_f_) at or below 100 nM [3]. Under optimized states, SERCAs transport 2 mol of Ca^2+^ across the SR membrane upon the hydrolysis of 1 mol of ATP [4–6]. Early estimates of the energy consumed by SERCAs in muscle under basal conditions were based on measurements of the rate of Ca^2+^ efflux from isolated SR vesicles and then the amount of ATP that would be required to reuptake that Ca^2+^ was calculated assuming an optimal Ca^2+^:ATP coupling ratio. This approach yielded values for the energetic cost of SR Ca^2+^ pumping of ~3.4–7% of resting muscle metabolism [7,8].

Although direct measurements are lacking, there is evidence to suggest that these values largely underestimate the energetic cost of SR Ca^2+^ pumping in resting skeletal muscle. For example, another approach that has been employed to determine the relative contribution of SR Ca^2+^ cycling to resting energy expenditure in skeletal muscle is to measure the decrease in energy expenditure following exposure of the muscle to chemicals that indirectly inhibit SERCAs by inhibiting Ca^2+^ leakage from SR Ca^2+^ release channels (CRCs). Using this approach with direct calorimetry, Chinet et al. [9] found that ~12-24% of resting energy expenditure in mouse soleus is related to Ca^2+^ cycling across the SR membrane. Similar experiments on mouse soleus and extensor digitorum longus (EDL) muscles showed that 18–22% of resting energy expenditure in both muscles is related to SR Ca^2+^ uptake [10]. However, CRC inhibition may underestimate the real contribution of SERCA activity to resting metabolic rate in skeletal muscle given that SERCA pumps themselves are a very significant pathway for leakage of Ca^2+^ out of the SR [11]. Furthermore, the aforementioned studies did not include proper control experiments to demonstrate that the CRC inhibitors employed (high Mg^2+^, 2,3-butanedione monoxime) specifically blocked calcium leakage from the SR.

Surprisingly, no study has used specific inhibitors of SERCAs to directly quantify the relative contribution of SR Ca^2+^ pumping to resting metabolic rate in intact skeletal muscle. Direct inhibition of SERCA avoids the problem of Ca^2+^ leaking through SERCA, but poses its own challenges, particularly increases in [Ca^2+^]_f_ which activates other cytosolic Ca^2+^-dependent ATPases, most notably the acto-myosin ATPase. Chinet et al. [9] attempted this approach but found it problematic to separate the effects of blocking SR Ca^2+^ uptake from the resulting rise in [Ca^2+^]_f_ and development of contracture on muscle energy expenditure. In order to accurately quantify the contribution of Ca^2+^ cycling to resting metabolic rate in skeletal muscle, SERCA activity must be inhibited directly in conjunction with myosin ATPase activity. This can be accomplished by employing stretch to eliminate filament overlap prior to inhibiting SERCA activity [12–14]. Here, we have attempted to: 1) validate the use of high Mg^2+^ to block SR Ca^2+^ leak in intact muscles in order to indirectly quantify the contribution of SR Ca^2+^ pumping to resting muscle metabolic rate; and 2) compare the use of both direct and indirect inhibition of SR Ca^2+^ cycling to accurately quantify the contribution of SR Ca^2+^ pumping to resting metabolic rate in mouse fast- and slow-twitch skeletal muscle using polarographic techniques. Despite preventing acto-myosin ATPase activity, we report difficulties measuring the metabolic cost of Ca^2+^ pumping using the direct approach to inhibit SERCA activity. When CRCs were inhibited with extracellular application of 10 mM MgCl_2_, thereby indirectly inhibiting SERCA activity, we measure the cost of maintaining Ca^2+^ homeostasis in resting muscle is 40-50% of the total muscle VO_2_.

## Materials and Methods

### Animal description and experimental conditions

A total of 44 sexually mature (6-7 months) male C57BL/6 mice were used in this study. Animals were housed 2-4 per cage in an environmentally controlled room with a standard 12:12 light/dark cycle and allowed access to food (Tekland 22/5 Rodent Diet, Harland-Tekland, Madison, WI) and water *ad libitum*. Animals were euthanized and the EDL, soleus or lumbrical muscles were carefully removed with tendons intact. Due to the time required for each experiment, only 1 muscle (EDL or soleus or lumbrical) could be assessed from each mouse. In total, 5 lumbrical muscles were used to determine whether treatment with high (10 mM) Mg^2+^ specifically blocks Ca^2+^ leakage from the SR and a total of 8 soleus and 7 EDL muscles were used to analyze the effects of indirect inhibition of SERCA by MgCl_2_. An additional 12 animals were used to test the effects of direct pharmacological inhibition of SERCA and 12 more animals were used to test the effects of stretch on VO_2_ in EDL and soleus to account for myosin ATPase activity. All animal procedures were approved by the Animal Care Committee at the University of Waterloo (AUPP 09-05) and all procedures were performed in accordance with the Canadian Council on Animal Care.

### Fluorescent Control Experiments with MgCl_2_

**Figure 1:**
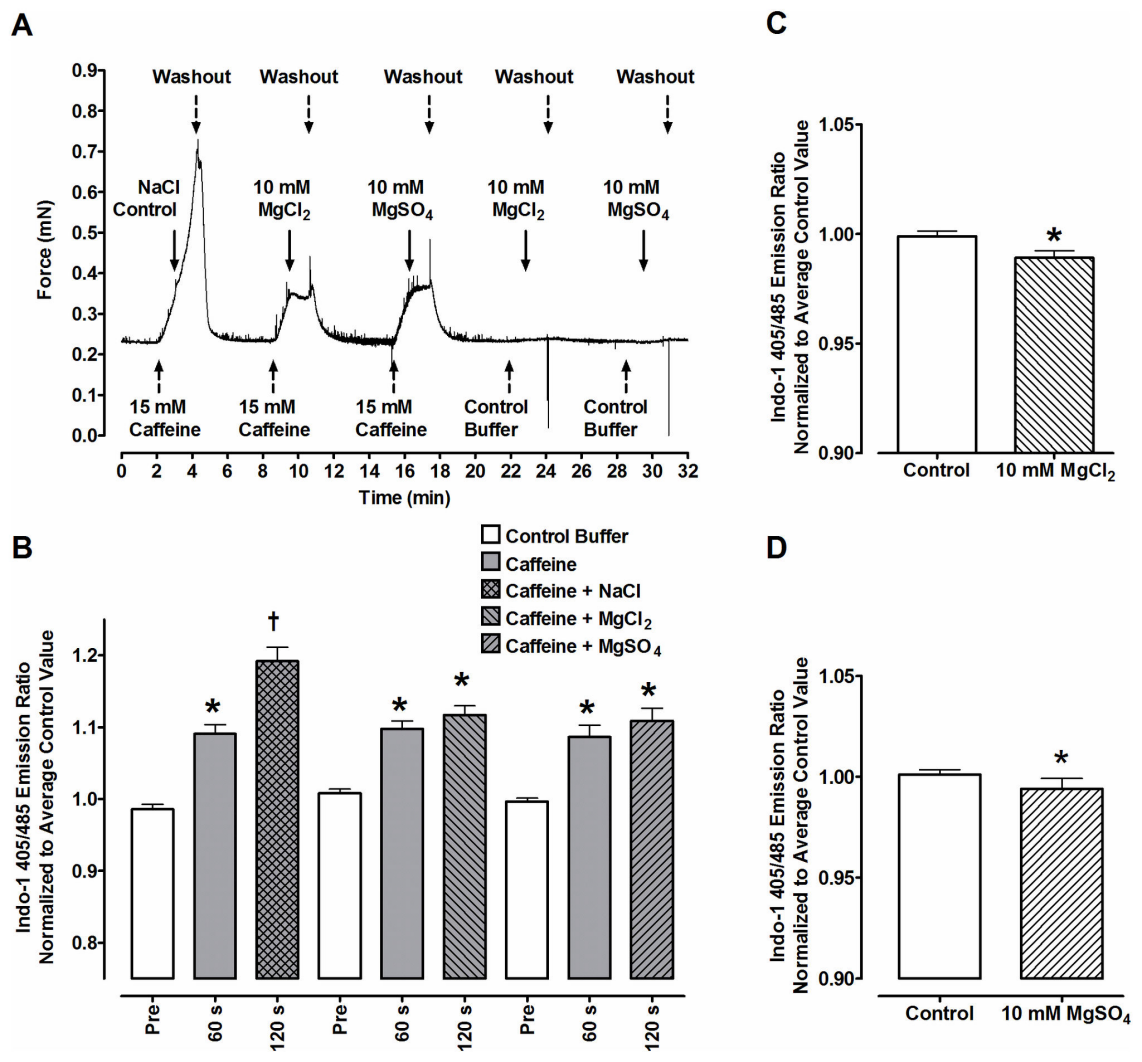
Effects of 10 mM Mg^2+^ on force and the indo-1 fluorescence ratio during caffeine-induced contracture. (A) The force response of an intact mouse lumbrical muscle loaded with AM-indo-1 in a circulating oxygenated buffer. The source of the buffer was alternated between a large reservoir (used during the initial 5 min incubation and during 2-3 min washout periods) and a small reservoir (used as site of Mg^2+^ or Na^+^ salt addition, and either contained or did not contain 15 mM caffeine as indicated). Switches between the two reservoirs are marked by the dashed arrows. Fluorescent measures (B) were collected immediately prior to the introduction of each solution originating from the small reservoir (labeled Pre and 60 s), and 60 s after the introduction of the Mg^2+^ or Na^+^ salt to the muscle (labeled 120 s). Bars correspond to the 1.0 s average of the 405/495 nm indo-1 emission ratio normalized to the average of the Pre ratios and are relative indicators of what is happening to the cytosolic Ca^2+^ concentration. *Significantly different than the preceding pre-switch value (P<0.05). †significantly different than all other values (P<0.05). Fluorescent responses of muscles to MgCl_2_ and MgSO_4_ in absence of caffeine are shown in panels C and D, respectively. Bars are analogous to 60 s and 120 s from Panel B *Significantly different than control value (P<0.05).

To demonstrate that treatment with 10 mM Mg^2+^ reversibly blocks Ca^2+^ release from the SR, experiments were performed on lumbrical muscles (n=5) loaded with the cell permeable AM form of the Ca^2+^-sensitive indicator, indo-1. Briefly, these muscles were isolated and mounted to a model 322C high speed length controller and a model 400A force transducer (Aurora Scientific Inc. (ASI)) at optimal length for twitch force production (L_o_) and incubated at 30^o^C in oxygenated (95% O_2_, 5% CO_2_) Ringer solution (121mM NaCl, 5mM KCl, 1.8mM CaCl_2_, 0.5mM MgCl_2_, 0.4mM NaH_2_PO_4_, 24mM NaHCO_3,_ 5.5 mM glucose and 0.1mM EDTA, pH 7.3) circulating, but not recycling, at 3.3 mL/min (1 bath change per 20 s). Muscles were then loaded with AM-indo-1 and background-corrected fluorescent signals were collected at 495 and 405 nm following 355 nm excitation as described in Smith et al. [15]. Following loading, force was continuously recorded at 10 Hz and stored for later analysis. The source of Ringers solution was alternated between a large and small reservoir serving as sources of control and drugged solutions, respectively, as demonstrated in Figure 1A. Caffeine (15 mM) was used to induce release of SR Ca^2+^ (reviewed in 16). After 60 s an addition of either 1.0 M NaCl, 1.0 M MgCl_2_ or 1.0 M MgSO_4_ was made to the small reservoir equal to 0.95% v/v of the remaining reservoir buffer, giving final [Mg^2+^] of 10 mM in the MgCl_2_ and MgSO_4_ trials. Again after 60 s, the source of Ringers solution was returned to the large reservoir and the muscle was allowed to re-equilibrate, which was defined as a stable baseline tension for 2-3 minutes, after which the cycle was repeated using different solutions.

### Oxygen consumption in isolated intact mouse skeletal muscles

The isolated EDL and soleus muscles were mounted in the sealed, jacketed chamber of the TIOX tissue bath system (Hugo Sachs Electronik-Harvard Apparatus, Germany) between a force transducer (F30 type 372, Harvard Apparatus) and a fixed hook. The chamber was then filled with Ringer solution which was pre-heated to 30^°^C and pre-oxygenated (95% O_2_, 5% CO_2_). The chamber was constantly stirred following filling. The muscle length was adjusted to achieve L_o_, with stimulation applied via flanking platinum plate electrodes and then the muscle was given 10 minutes to equilibrate inside the chamber prior to initiating data collection.

**Figure 2:**
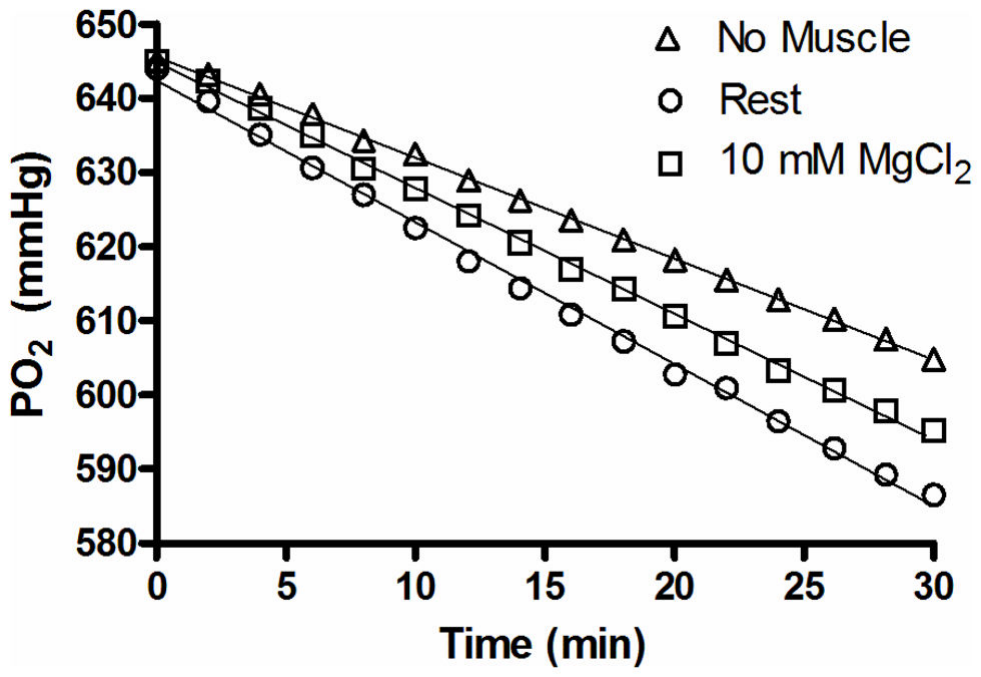
Representative raw tracing of PO_2_ decline over 30 minutes at 30^°^C for an O_2_ leak trial (No Muscle, Δ), a resting muscle VO_2_ trial (Rest, ○) and a high MgCl_2_ trial (10 mM MgCl_2_, □) for a soleus muscle.

VO_2_ measurements were performed using a Clarke type PO_2_ electrode (model 1302) mounted in the chamber and calculated by multiplying the measured drop in PO_2_ with time by the solubility of oxygen in Ringer solution at 30^°^C and the chamber volume (12.7 mL). The solubility of oxygen at 30^°^C was calculated to be 0.001203 M/atm. Blank trials were done at the beginning and at the end of daily data collection to account for the rate of oxygen loss from the empty chamber (i.e. no muscle). The average daily rate of oxygen leak is then subtracted from the oxygen loss in the presence of the muscle to give the muscle VO_2_. A sample PO_2_ tracing of a leak (No Muscle), rest, and 10 mM MgCl_2_ trial is depicted in Figure 2. The average rate of oxygen leak (µL/s) over the course of the experiment was found to be 0.0071 ± 0.0002 with a coefficient of variation of 6.4%. Muscle VO_2_ is reported relative to muscle wet weight (µL/g wet weight/s).

PO_2_ data were collected during each of 3 separate experimental trials at 30^°^C designed to quantify resting muscle VO_2_ and SERCA pump energetics. The PO_2_ in the bath (~620 mmHg) should enable adequate diffusive oxygen supply to support resting muscle metabolism of mouse soleus and EDL [17]; however, to prevent the formation of hypoxia in the core of the muscle, all experiments were terminated before the PO_2_ of the Ringer solution fell below 580 mmHg. The decrease in PO_2_ of the Ringer solution was recorded in the presence of a resting muscle at L_o_ during serial 30 minute incubations in normal Ringer solution (0.5 mM MgCl_2_), Ringer solution with an elevated concentration of MgCl_2_ (10 mM), and normal Ringer solution once again to verify muscle viability.

Since elevated [Mg]_i_ and reduced [Ca^2+^]_i_ would be highly inhibitory to myosin ATPase activity due to a lack of thin filament activation [18], a subset of experiments were performed using stretch (1.42 L_o_) to prevent thick and thin filament overlap to determine the metabolic cost of crossbridge cycling in resting EDL and soleus muscles.

**Figure 3:**
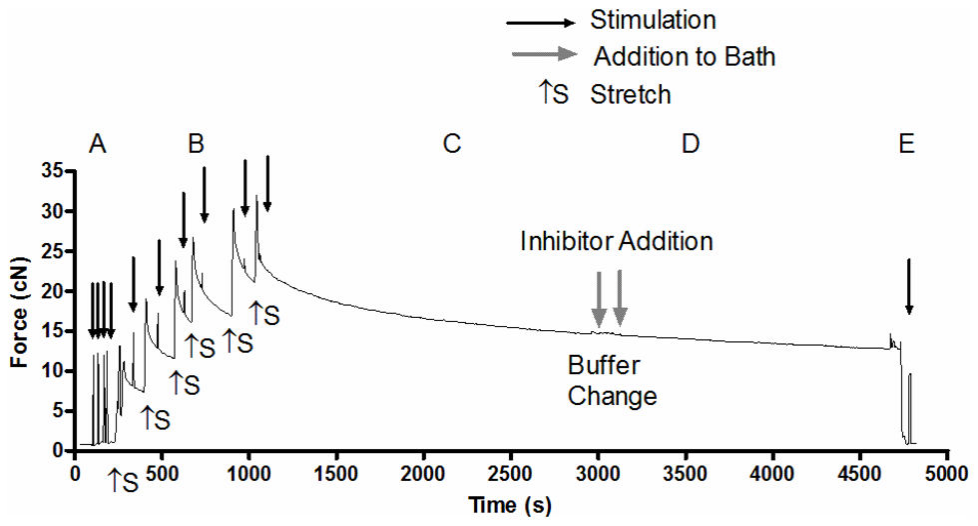
Representative tracing of muscle force for one EDL muscle experiment. (A) Twitch force at L_o_ (no CPA); (B) twitch force at lengths >L_o_ (no CPA); (C) passive force throughout the period of pre-stretch (no CPA); (D) passive force throughout the CPA trial; and (E) twitch force at L_o_ following the CPA trial. Thin black arrows represent muscle stimulation (pulse duration, 0.2 ms; voltage, 10 V). Thick grey arrows indicate when fresh buffer and CPA were added. ↑S indicates where the incremental stretches were applied to the muscle.

To test the efficacy of using a pharmacological inhibitor to eliminate the metabolic cost of SERCA activity, muscle VO_2_ was recorded at 1.42 L_o_ in the presence of cyclopiazonic acid (CPA), a highly specific and potent SERCA inhibitor [19–21], prepared at stock concentration of 40 mM in DMSO with final bath concentrations of 10, 40 and 160 µM CPA. A representative force tracing illustrating the protocol is shown in Figure 3.

### Muscle homogenates

For Western blotting, homogenates were prepared with whole soleus and EDL muscles diluted 19:1 (vol/wt) in an ice-cold buffer containing 250 mM sucrose, 5 mM HEPES, 10 mM NaN_3_, and 0.2 mM PMSF (pH 7.5) by using a handheld glass homogenizer (Duall 20, Kontes). Total protein concentration of the homogenates was measured by the method of Lowry, as modified by Schacterle and Pollock [22].

### Preparatation of SERCA1a and SERCA2a protein standards

SR membrane fractions were isolated from white portions of gastrocnemius muscles and whole heart from 3–4 month old Sprague-Dawley rats as described in earlier publications [23,24]. Subsequently, SERCA1a and SERCA2a were immunoprecipitated from the gastrocnemius and heart fractions, respectively [25]. The purified protein (SERCA1a or SERCA2a) was confirmed through polyacrylamide gel electrophoresis and Ponceau (Sigma) staining. The purified protein was also differentiated with different amounts of BSA standard through SDS-PAGE, transferred to PVDF membrane, stained with Ponceau, and quantified using a bio-imaging system and densitometric analysis with the GeneTools software (Syngene).

### Western blotting

Western blotting was performed to determine the content and relative proportion of SERCA1a and SERCA2a in EDL and soleus muscles. Homogenates (SERCA1a, 0.075 and 0.25 µg total protein for EDL and soleus, respectively; SERCA2a, 4 and 1 µg total protein for EDL and soleus, respectively) were applied to 7% polyacrylamide gels, and proteins were separated using standard SDS-PAGE protocols [26] and then transferred to polyvinlyidene difluoride (PVDF) membranes (BIO-RAD). After blocking with a 5% skim milk suspension, the membranes were incubated for 1 hr with either anti-SERCA1a monoclonal antibody A52 [27] or anti-SERCA2a antibody 2A7-A1 (Affinity Bioreagents Inc.). Then, after washing in Tris-HCl, pH 7.5, 150 mM NaCl, 0.1% Tween 20 (TBS-T), the membranes were treated with horseradish peroxidase-conjugated anti-mouse secondary antibody (Santa Cruz Biotechnology). Membranes were washed in TBS-T, and the signals were detected with an enhanced chemiluminescence kit (GE Healthcare) using a bio-imaging system and densitometric analysis was performed using the GeneTools software (Syngene). For quantification of SERCA1a and SERCA2a, standard curves were plotted (density *versus* known amounts of pure protein [SERCA1a or SERCA2a]) and the amounts of SERCA1a or SERCA2a in a given sample (molecules/fg total protein) were determined from standard curves on the same Western blot [11].

### Statistical analyses

A two-way repeated measures ANOVA was employed to analyze the muscle type specific (soleus vs. EDL) changes in VO_2_ in response to different concentrations of MgCl_2_ (0.5 mM (rest) vs. 10 mM (high MgCl_2_) vs. 0.5 mM (washout)). Student’s *t* test was utilized for all other comparisons. The significance level was set at 0.05, and when appropriate, a Newman-Keuls post hoc test was used to compare specific means. Values are means ± SE.

## Results

### Fluorescent Control Experiments with MgCl_2_

The addition of 15 mM caffeine to the bathing solution of the intact muscles resulted in a progressive, reversible increase in tension. Tension continued to increase following exposure to the control addition of 0.95% v/v 1.0 M NaCl (Figure 1A). This effect was similar when 1.0 M NaCl was substituted with 2.0 M NaCl, water, or no addition was made at all (not shown). However, when exposed to 10 mM MgCl_2_ or MgSO_4_ in the presence of caffeine, the rate of tension development was significantly slower than the control NaCl experiments (as in Figure 1A), an effect which also occurred with 15 mM caffeine exposure when the muscle was pre-exposed to either 10 mM MgCl_2_ or MgSO_4_ (P<0.05, data not shown). These changes were consistent with the changes seen in [Ca^2+^]_i_ indicated by the indo-1 fluorescence ratio (Figure 1B). Namely, [Ca^2+^]_i_ increased following caffeine exposure, but this increase was attenuated with the addition of either 10 mM MgCl_2_ or MgSO_4_. Similarly, exposure to either MgCl_2_ or MgSO_4_ alone resulted in a significant decrease in the resting [Ca^2+^]_i_ (Figure 1C and D). These results demonstrate that Mg^2+^ is able to quickly enter intact muscle and inhibit Ca^2+^ release from the SR, thus the assumption that Mg^2+^ can inhibit SERCA indirectly by reducing the amount of Ca^2+^ seen by SERCA is tenable.

### Resting muscle VO_2_

**Figure 4:**
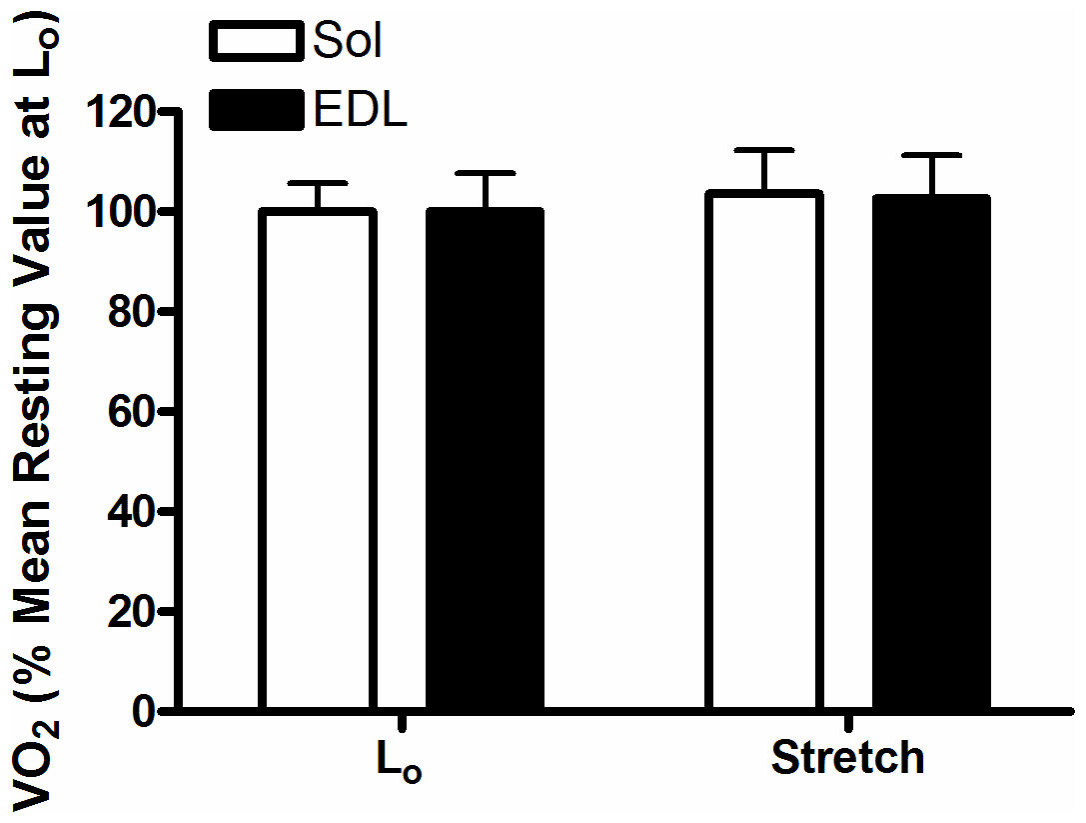
Resting oxygen consumption of EDL and soleus muscles at optimal length (L_o_) compared to oxygen consumption during stretch trial (no sarcomere overlap) at 30^°^C for 30 minutes. VO_2_ is expressed relative to average resting VO_2_ at L_o_. Values are mean ± SE (n=6).

**Figure 5:**
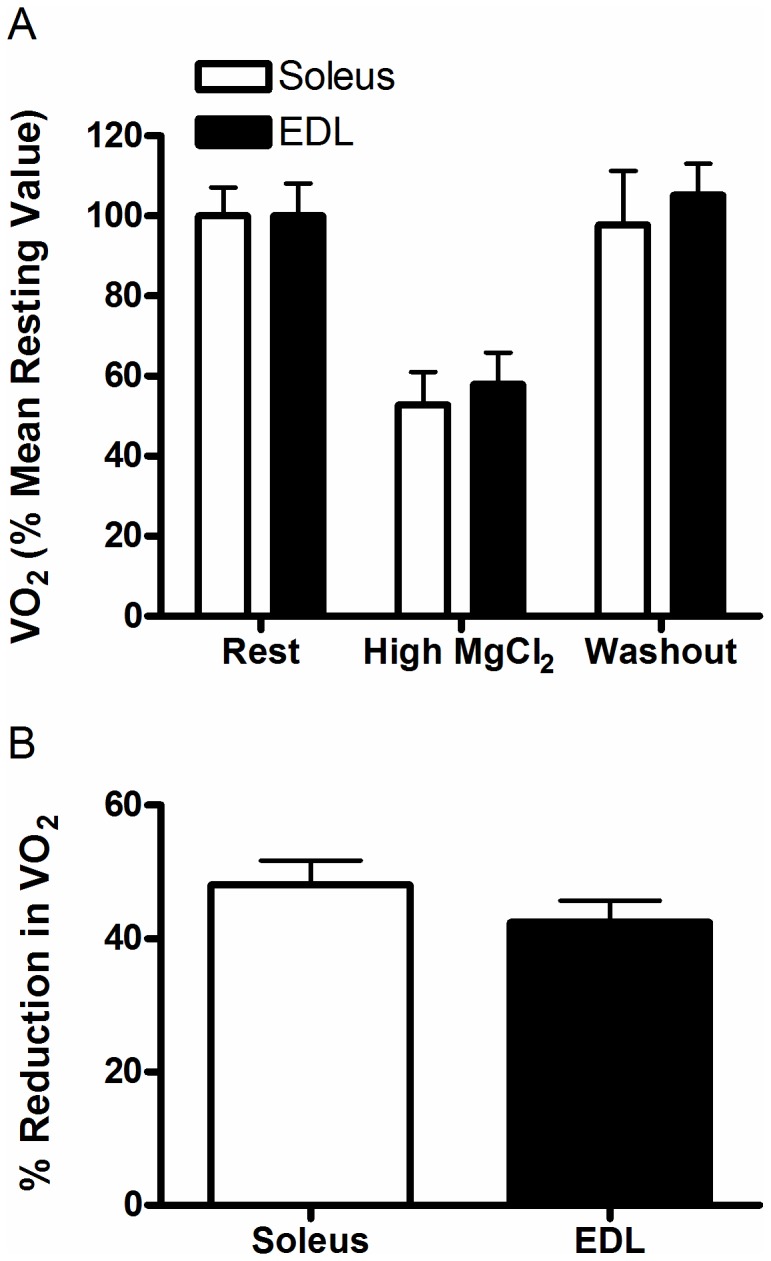
Effects of 10 mM MgCl_2_ on resting VO_2_ of isolated soleus and EDL muscles. (A) Resting VO_2_ of isolated soleus (n=14) and EDL (n=13) muscles during serial 30 minute incubations in solutions containing 0.5 (Basal and Washout) or 10 (High MgCl_2_) mM MgCl_2_. Incubation with 10 mM MgCl_2_ reversibly reduced resting VO_2_ in both soleus and EDL muscles (Main effect, High MgCl_2_ < Basal, Washout; P<0.001). (B) Percent reduction in resting VO_2_ of isolated soleus and EDL muscles induced by incubation in high (10 mM) MgCl_2_.

The rates of oxygen consumption of the soleus and EDL muscles were measured for 30 minutes at 30^°^C (n=20 soleus, 19 EDL). The average resting VO_2_ (µL/g wet weight/s) at 30^°^C was ~20% higher (P<0.05) in soleus (0.326 ± 0.02) compared with EDL (0.261 ± 0.02). Pre-stretching the muscles had no effect (P>0.05) on resting VO_2_ of either soleus or EDL muscles, with stretched VO_2_ equal to 104 ± 9 and 102 ± 10% of resting VO_2_ at L_o_, respectively (n=6; Figure 4). Resting VO_2_ of both soleus and EDL was stable with time as the rates measured from 0–30 min (Rest) of incubation were not different (P>0.05) than measurements taken from 60–90 min (Washout) of incubation (Figure 5A).

### Oxygen cost related to SR Ca^2+^ pumping in EDL and soleus muscles

#### a) Indirect inhibition

The O_2_ costs attributable to Ca^2+^ pumping were 0.126 ± 0.02 and 0.107 ± 0.02 µL/g wet weight/s in soleus and EDL respectively. The percent contribution of Ca^2+^ pumping by SERCAs to resting VO_2_ was calculated by dividing the difference between the VO_2_ values of the resting trial and the 10 mM MgCl_2_ trial by resting VO_2_ for both soleus and EDL (Figure 5A). The relative (%) reduction in muscle VO_2_ in response to 10 mM MgCl_2_ was not different between EDL (42.4±3.2) and soleus (48.0±3.7) muscles (P=0.28; Figure 5B). The addition of MgCl_2_ did not affect the rate of O_2_ loss from the chamber in the absence of muscle (data not shown).

#### b) Direct inhibition

**Figure 6:**
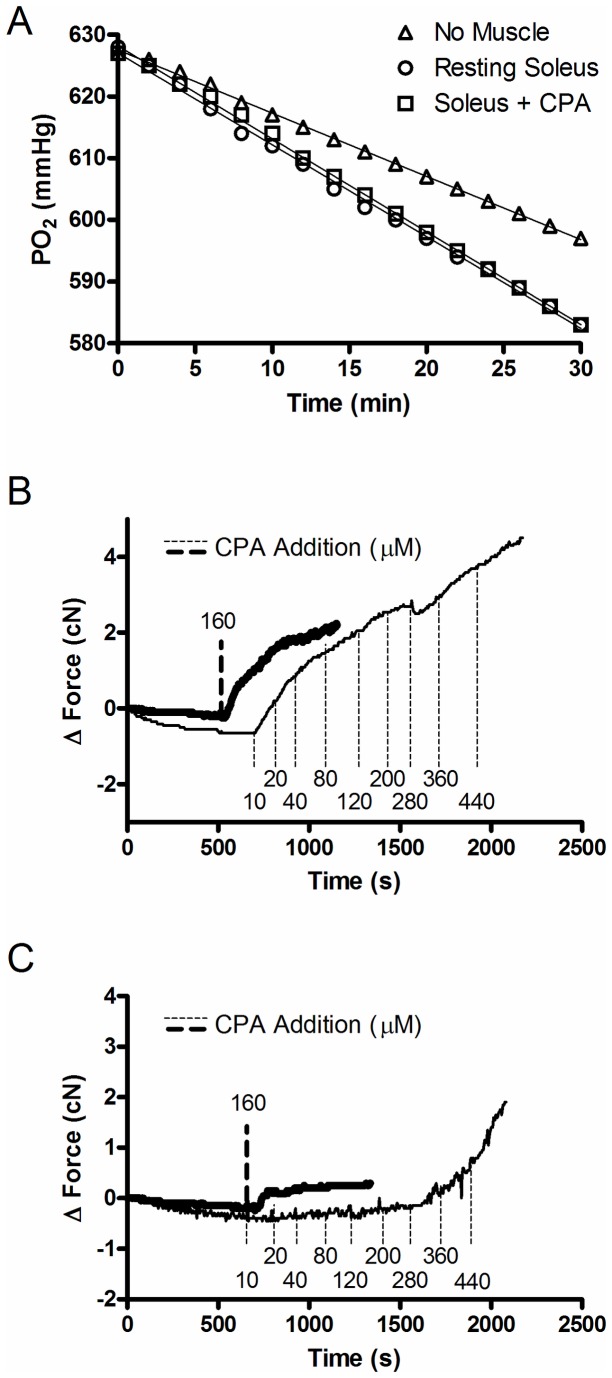
Effects of CPA on resting VO_2_ and baseline tension of isolated soleus and EDL muscles. (A) Representative raw tracings of PO_2_ decline over 30 minutes at 30^°^C for an O_2_ leak trial (No Muscle, Δ), a resting muscle VO_2_ trial (Rest, ○) and a 160 µM CPA trial (□) for a soleus muscle and muscle force for two different EDL (B) and soleus (C) muscles showing changes in resting tension at L_o_ following a single addition of 160 µM CPA (thick lines) and serial dilutions of CPA (thin lines) with cumulative CPA concentrations enumerated.

Sample PO_2_ tracings of leak (No Muscle), rest, and 160 µM CPA trials for a soleus muscle are depicted in Figure 6A. Incubation of pre-stretched isolated soleus and EDL muscles with CPA (10-160 µM) did not result in a reduction of resting muscle VO_2_ (n=2 per concentration; data not shown). Importantly, there was no increase in resting tension following the addition of CPA in the stretched muscle indicating that myosin ATPase was not activated during the addition (see Figure 3). To test the possibility that CPA did not effectively inhibit SERCA activity in the whole muscle preparations that were used for these studies, we also performed some CPA experiments on muscles that were maintained at L_o_ and monitored changes in resting tension for up to 30 min following CPA addition. Following addition of 160 µM CPA, soleus resting tension increased slowly from baseline and started to plateau at ~2.45 cN within 10 min (Figure 6C). Therefore in soleus, CPA induced a small contracture equaling ~16% of maximal tetanic force. In EDL, the CPA-induced contracture was even smaller, plateauing at ~0.5 cN (Figure 6B). Serial additions of CPA (up to 440 µM) caused a progressive increase in muscle force and prevented the force from reaching a plateau over the course of the experiments. Additionally, twitches had longer time to peak tension and slower half relaxation times with increasing doses of CPA and these effects were more pronounced in the soleus compared with the EDL (data not shown). Importantly, muscles were able to fully relax after twitch stimulation. Collectively, these results demonstrate that CPA (up to 160 µM) inhibits SERCA activity in resting intact mouse soleus only marginally and the effects in resting EDL are negligible.

### SERCA density and isoform distribution in soleus and EDL muscles

**Figure 7:**
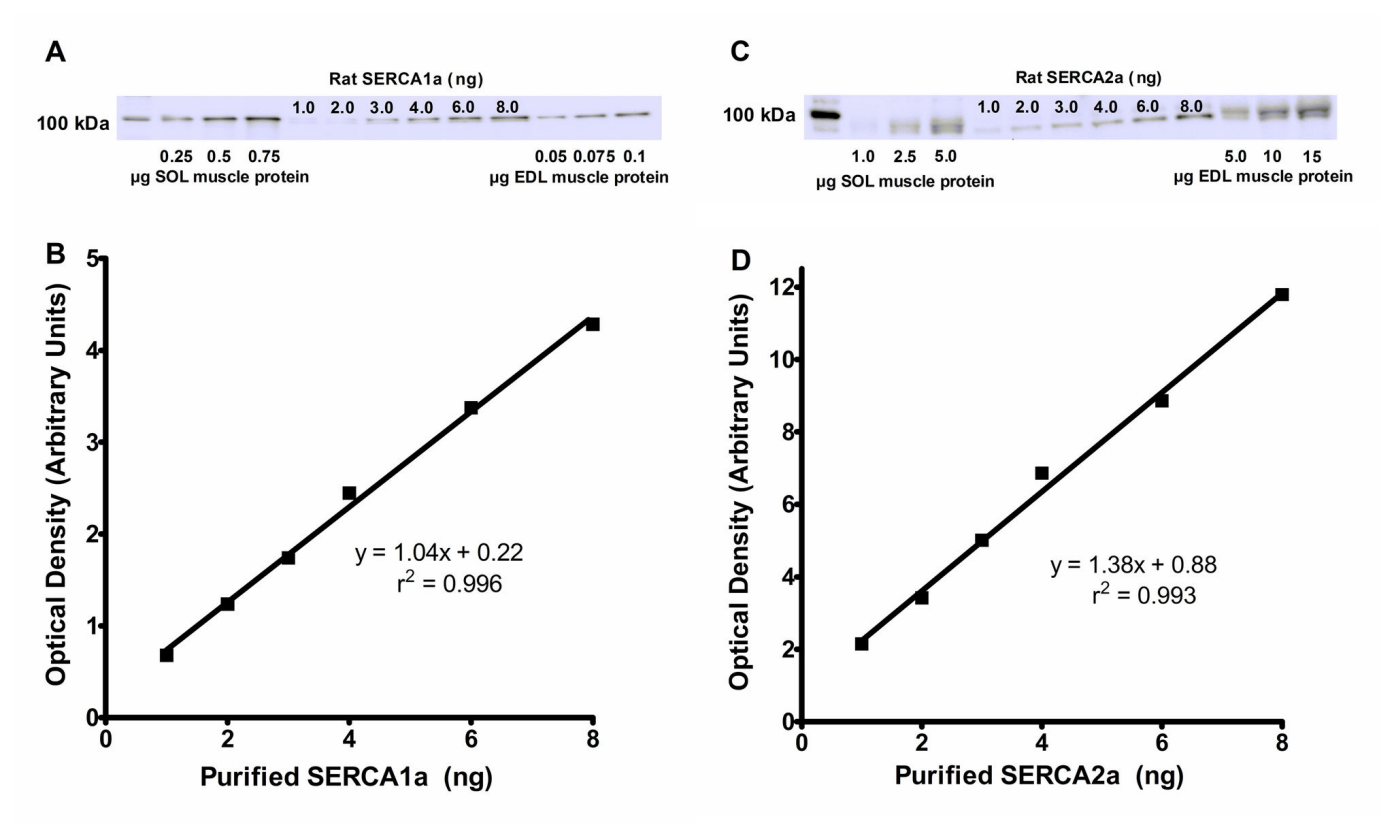
Quantification of SERCA1a and SERCA2a in mouse EDL and soleus muscle homogenates. Protein from mouse EDL and soleus whole muscle homogenates (0.05-15.0 µg total protein, as indicated) and 1.0-8.0 ng purified SERCA1a (prepared from rat skeletal muscle) or SERCA2a (prepared from rat heart) were separated on a 7% SDS-PAGE gel and SERCA1a (A) or SERCA2a (C) were detected by Western blotting using the A52 and 2A7-A1 antibodies, respectively. Density of band for purified SERCA1a (B) and SERCA2a (D) was plotted against amount of protein. The amount of SERCA1a and SERCA2a in EDL and soleus homogenates was determined from intensity of bands that fell within the linear range for samples of purified SERCA1a and SERCA2a on the same gel.

The content and relative distribution of SERCA pump isoforms (SERCA1a + SERCA2a) were compared in EDL and soleus by quantitative Western blotting as described under “Materials and Methods” (e.g. Figure 7). The amount of SERCA1a (mg/g total muscle protein) was ~5.2-fold higher (P<0.05) in EDL (46 ± 4.3) compared with soleus (8.9 ± 0.5) and the amount of SERCA2a (mg/g total muscle protein) was not significantly different (p=0.08) between EDL (0.51 ± 0.07) and soleus (0.65 ± 0.18). The number of SERCA1a and SERCA2a pumps per fg total muscle protein was 254 ± 23 and 2.8 ± 0.4, respectively, in EDL and 49 ± 3 and 3.6 ± 1, respectively, in soleus. Therefore, the ratio of SERCA1a:SERCA2a was ~91:1 in EDL compared with ~14:1 in soleus and in total there were ~4.9-fold more SERCA pumps (SERCA1a + SERCA2a) in EDL than soleus.

## Discussion

We hypothesized that previous approaches [9,10] using CRC blockers to inhibit SR Ca^2+^ release and indirectly inhibit SERCA activity by lowering [Ca^2+^]_f_ would underestimate the ATP turnover by SERCAs in resting muscle. Therefore, in this study we attempted to directly inhibit SERCA activity pharmacologically using CPA to more accurately quantify the contribution of SERCA activity to resting energy expenditure in mouse skeletal muscle. Surprisingly, this treatment did not result in any decline in muscle VO_2_ indicating the futility of this approach, at least with whole muscle preparations. However, consistent with earlier findings [9,10], when we inhibited SR Ca^2+^ release using a high concentration (10 mM) of MgCl_2_ and thus indirectly inhibited SERCA activity, we showed that the relative (%) contribution of Ca^2+^ dependent ATP turnover to resting metabolic rate is substantial (i.e. ~40-50%) and not different between EDL and soleus.

The lack of effect in this study of CPA on muscle VO_2_ could be due to either activation of non-myosin, Ca^2+^-dependent ATP consuming processes, or incomplete inactivation of SERCA. CPA is known to be cell permeable and the concentrations used in this study exceeded those known to maximally inhibit SERCA activity in skinned fiber preparations [28]. However, Baudet et al. [29] found that 100 µM CPA could not fully inhibit SERCA activity in a multicellular cardiac preparation. Similarly, based on the magnitude of effects of CPA on twitch relaxation and resting tension observed in this study, we conclude that SERCA Ca^2+^ transport activity was only partially inhibited by 160 µM CPA, which likely accounts primarily for the lack of effect of CPA on resting muscle VO_2_ in both soleus and EDL. Nevertheless, SERCA activity was clearly reduced in the presence of CPA, as evidenced by mild contracture at least in soleus, yet resting VO_2_ in pre-stretched muscles was unaltered under those conditions. One possible explanation for this apparent discrepancy is that CPA-induced reductions in ATP consumption by SERCAs were offset by equivalent increases in Ca^2+^-dependent mitochondrial respiration [30], or Ca^2+^-calmodulin dependent protein kinase activity, resulting in higher metabolic rate due to cycling of phosphorylation/dephosphorylation reactions [2,31]. Furthermore, even though the CPA-induced contracture was barely detectable in EDL (Figure 6B), that should not be taken to indicate that only a few SERCA pumps were inhibited in EDL under those conditions. At the low rates of Ca^2+^-ATPase activity required to maintain Ca^2+^ homeostasis in resting muscle, any SERCA pump that is not inhibited should be able to increase its rate of Ca^2+^ pumping to compensate for those pumps which are blocked, the net result being no change in total ATP consumption by SERCAs.

Indirect inhibition of SERCAs by 10 mM MgCl_2_ to prevent Ca^2+^-release through the Ca^2+^-release channel was effective in reducing muscle VO_2_. We showed that 10 mM Mg^2+^ specifically blocked Ca^2+^ release from the SR during caffeine exposure and under resting conditions (i.e. in the absence of caffeine), thus lowering [Ca^2+^]_i_. However, high extracellular Mg^2+^ has been shown to change the surface charge potential and shift the contraction threshold of frog skeletal muscle fibers to less negative potentials, mimicking hyperpolarization [32], which would also reduce Ca^2+^ leak out of the SR, and thereby reduce ATP consumption by SERCA pumps. Therefore, this represents a plausible alternative explanation for our results but one that would not change our conclusions regarding the contribution of SERCA pumps to resting muscle metabolic rate. In either case, our results support the view that the effects of 10 mM Mg^2+^ on muscle VO_2_ were due to lower ATP consumption by SERCA pumps. Indeed, our results indicate that ATP consumption by SERCAs is responsible for ~42 and 48% of resting metabolic rate in EDL and soleus respectively, a quantity that is approximately double that previously reported [9,10]. Compared with those studies, it is unclear why muscle VO_2_ was reduced to a greater extent in our study following exposure to high Mg^2+^. Interestingly, Chinet et al. [9] showed that under ‘near-basal’ conditions, in which Ca^2+^ release was slightly enhanced at levels that were subthreshold-for-contracture, Ca^2+^-dependent energy expenditure accounted for ~40% of total metabolic rate in intact mouse soleus. If the muscles were slightly depolarized under our experimental conditions, then perhaps our measurements were actually made under ‘near-basal’ conditions or perhaps differences in mouse strain could explain differences between the studies. Consistent with our results, Dulloo et al. [10] found that the relative (%) contribution of Ca^2+^ dependent ATP turnover to resting metabolic rate was equal between EDL and soleus. Since this reduction in VO_2_ was completely reversible and VO_2_ was stable for 90 minutes (Figure 5A), we can be confident that diffusive O_2_ supply in these experiments was adequate to support resting metabolism over the course of the experiment and our results are not due to a progressive decline in muscle VO_2_.

In this study, we measured rates of oxygen consumption at 30^o^C in resting mouse EDL and soleus using the TIOX tissue bath system. With the exception of at least one other study [33], our finding that resting VO_2_ was ~20% higher in soleus compared with EDL is in agreement with most previous studies [10,13,34]. The reduction in muscle VO_2_ we observed after increasing the concentration of Mg^2+^ (i.e. the VO_2_ attributable to SERCA activity) corresponds to an absolute ATP turnover rate by SERCAs at the whole muscle level of 73.0 ± 12.1 nmol/g/s (EDL) and 82.0 ± 11.2 nmol/g/s (soleus). Assuming that [Ca^2+^]_f_ is constant in resting skeletal muscle, basically 2 main factors determine the ATP turnover rate by SERCAs in resting muscle: 1) the rate of Ca^2+^ leakage out of the SR and 2) the stoichiometry of Ca^2+^ uptake by SERCAs (i.e. Ca^2+^/ATP ratio). Measurements in mechanically skinned fibres from rat EDL and soleus indicate that under normal resting conditions, Ca^2+^ leaks out of the SR at approximately the same absolute rate in both muscles [35,36]. CRCs and SERCAs represent the major pathways for Ca^2+^ leakage from the SR, although SERCAs appear to be of primary importance in this regard [11,35,36]; therefore, results from our MgCl_2_ experiments may even underestimate the true contribution of SERCAs to resting muscle metabolic rate. It is well known that the density of CRCs is ~2-3-fold higher in EDL than soleus [37,38] and the SERCA density is ~3-8-fold higher in EDL than soleus [11,39,40]. In this study, we found that mouse EDL contained ~4.9-fold more SERCA pumps than soleus. Therefore, it might seem surprising that the rate of Ca^2+^ leakage from the SR is the same in EDL and soleus; however, Murphy et al. [11] have shown that the high content of calsequestrin in EDL muscle fibres helps to keep the free [Ca^2+^] in the SR at sufficiently low levels to prevent high rates of Ca^2+^ leakage through the high density of SERCA pumps. Assuming that rat and mouse fibres are comparable and the SR Ca^2+^ leak rates are the same in mouse EDL and soleus, the similar rates of ATP turnover by SERCAs we observed in EDL and soleus would therefore suggest that the efficiency of Ca^2+^ uptake by SERCAs in soleus and EDL is also similar, at least under resting conditions.

A 2:1 ratio of Ca^2+^ transport to ATP hydrolysis by SERCAs is considered to be optimal as it corresponds to the stoichiometry of two Ca^2+^ binding sites and one ATP binding site on each SERCA pump [41,42]. However, our values for ATP turnover by SERCAs strongly suggest that the Ca^2+^:ATP coupling ratio is actually much lower than 2:1 under physiological conditions. If the coupling ratio was 2 Ca^2+^: 1 ATP, the rate of Ca^2+^ uptake (which is equal to the rate of SR Ca^2+^ leakage in resting muscle) would be ~146 nmol/g/s in EDL and ~164 nmol/g/s in soleus which is ~10-11-fold higher than what has been measured in mechanically skinned EDL and soleus fibers from the rat [35,36,43]. Taking into account the ATP turnover rates we measured in this study and the SR Ca^2+^ leak rate reported by Lamb and Cellini [35,36,43] for rat EDL (which would be equal in soleus), we can calculate coupling ratios of ~0.23 Ca^2+^/1ATP in EDL and ~0.20 Ca^2+^/1ATP in soleus. Although the calculated coupling ratios for mouse EDL and SOL are similar to measurements made in SR preparations isolated from various rodent and canine muscles [44–46], we are likely underestimating the true SR Ca^2+^ leak rates in EDL and soleus under the experimental conditions employed in this study as Ca^2+^ leak out of the SR through SERCA could not be prevented by high Mg^2+^ treatment [11]. Therefore, these calculations also likely underestimate the coupling ratios in EDL and soleus. Furthermore, the amount of slippage/leakage of SERCA pumps increases with increasing temperature [47] and [ADP] [35]. Compared with the skinned fiber study by Lamb and Cellini [35,36,43], both the experimental temperature (23 vs. 30^°^C) and the [ADP] concentration in the fibers (~0.1 µM vs. ~10 µM) were higher in the present study. Higher coupling ratios have been reported for rabbit skeletal muscle preparations and, in contrast with our calculations, the Ca^2+^/ATP ratio was higher in slow-twitch muscle SR vesicles (i.e. 0.98 Ca^2+^/1ATP) compared with fast-twitch skeletal muscle SR vesicles (i.e. 0.37 Ca^2+^/1ATP) [48]. The coupling ratio differences found between slow and fast muscle were attributed to differences in SERCA isoform expression since SR vesicles from rabbit fast-twitch skeletal muscle only expressed SERCA1a whereas vesicles from rabbit slow-twitch skeletal muscle expressed preferentially SERCA2a [48]. In contrast, we found that over 90% of the SERCA molecules found in both mouse EDL and soleus were SERCA1a which could explain why the calculated coupling ratios for each muscle are closer to the ratios that were reported for rabbit fast-twitch skeletal muscle vesicles in the study by Reis et al. [48].

In adult C57BL/6 mice, the EDL contains >90% type II fibers (mostly IIB) and <10% type I fibers whereas the soleus contains ~50% type I and type II fibers (mostly IIA) [49,50]. Our results do not identify the contribution of the different fiber types to muscle VO_2_ or the relative contribution of SERCA activity to the metabolic rate of the different fiber types. It is worth noting that Murphy et al. [11] reported that virtually all of the SERCA1a found in rat soleus muscles were present in the fast-twitch fibers. Interestingly, the relative abundance of SERCA1a and SERCA2a mRNA levels in mouse (strain 129sV/Swiss 50/50) soleus also appears to match closely the fiber type distribution (i.e. 58% SERCA2 and 42% SERCA1) [40]. Importantly, the protein contents of the different SERCA isoforms in mouse soleus have not been quantified previously. In this study, SERCA1a and SERCA2a were quantified by comparing the Western blot band intensities of mouse muscle samples relative to purified proteins obtained from rat muscles. Although it is quite possible that the SERCA antibodies were differentially sensitive to the mouse and rat SERCA proteins, which would have caused an unknown level of error in the quantification, based on our results showing that over 90% of the SERCA molecules found in mouse soleus were SERCA1a, it is likely that SERCA1a is present in at least some type I mouse soleus fibers. Future studies should examine SERCA isoform protein expression in individual fiber types from mouse muscles to test this postulate.

It is well known that SERCAs play an important role in thermogenesis [30,47] but SERCAs may also play an important role in the regulation of whole body energy balance [51]. Assuming that skeletal muscle accounts for 30% of basal metabolic rate in mice [2] and that the results of our current study can be extrapolated to all mouse muscles at physiological temperature, SERCA activity can explain ~12-15% of whole animal resting VO_2_. It is also well known that SERCAs contribute ~30-40% to the energy cost associated with muscle contraction (for review, see 52). Therefore, given that basal metabolic rate and physical activity account for ~60 and 30% of total daily energy expenditure, respectively [2,53], and that skeletal muscle accounts for ~90% of the increased energy requirements associated with physical activity [2], SERCAs in skeletal muscle may explain ~15-20% of whole body total daily energy expenditure.

In summary, the results from this study support the conclusion that ATP consumption by SERCAs is responsible for 40-50% of resting metabolic rate in both mouse EDL and soleus muscles at 30^°^C. These results have important implications for the basic understanding of muscle energetics and suggest that the SERCA pump in skeletal muscle represents an important control point for energy balance regulation and a potential target for metabolic alterations to oppose obesity and other metabolic disorders.
